# Does a priming warm-up influence the incidence of $\dot{V}O_{2pl}$ during a ramp test and verification phase?

**DOI:** 10.1371/journal.pone.0313698

**Published:** 2025-01-08

**Authors:** JianBo Qiao, Paul Rosbrook, Daniel K. Sweet, Riana R. Pryor, David Hostler, David Looney, J. Luke Pryor

**Affiliations:** 1 Center for Research and Education in Special Environments, Department of Exercise and Nutrition Sciences, University at Buffalo, Buffalo, NY, United States of America; 2 United States Army Research Institute of Environmental Medicine (USARIEM), Natick, MA, United States of America; UTMB: The University of Texas Medical Branch at Galveston, UNITED STATES OF AMERICA

## Abstract

**Objective:**

This study compared the effects of two different warm-up protocols (normal vs. priming) on the oxygen plateau ($\dot{V}O_{2pl}$) incidence rate during a ramp test. It also compared the cardiopulmonary responses during the ramp test and subsequent verification phase.

**Methods:**

Eleven recreational cyclists performed two experimental visits. The first visit required a normal warm-up (cycling at 50 W for 10 min) followed by the ramp test (30 W^.^min^-1^) and supramaximal verification phase with 30 min rest between tests. The second visit required a priming warm-up (cycling at 50 W for 4 min increasing to 70% difference between the gas exchange threshold [GET] and maximum work rate [WR_max_] for 6 min) followed by the same protocol as in the first visit. Physiological responses were collected during the exercise and compared. Oxygen kinetics ($\dot{V}O_{2}$ Kinetics) and $\dot{V}O_{2pl}$ incidence rate were determined during the ramp tests for both visits.

**Results:**

As planned, following the warm-up the priming visit experienced greater physiological response. However, the incidence rate of $\dot{V}O_{2pl}$ during the ramp test was the same between visits (73%), and maximal oxygen uptake was not different between visits after the ramp test (normal, 4.0 ± 0.8; primed, 4.0 ± 0.7 L·min^−1^, *p* = 0.230) and verification phase (normal, 3.8 ± 0.6; primed, 3.8 ± 0.7 L·min^−1^, *p* = 0.924) using a Holm-Bonferroni correction for controlling family-wise error rate. $\dot{V}O_{2}$ Kinetics were not different between visits during the ramp test (normal, 10.8 ± 1.1; primed, 10.8 ± 1.2 mL·min^−1^·W^-1^, *p* = 0.407). The verification phase confirmed $\dot{V}O_{2max}$ in 100% for both the normal and priming visits.

**Conclusion:**

Our hypothesis that a priming warm-up facilitates the incidence rate of $\dot{V}O_{2pl}$ during a ramp test is not supported by the results. The verification phase remains a prudent option when determining a ‘true’ $\dot{V}O_{2max}$ is required.

## Introduction

Maximal oxygen uptake ($\dot{V}O_{2max}$) represents the physiologic upper limit of intaking and transporting oxygen for use in skeletal muscle mitochondria associated with substrate oxidation [1]. There is great clinical and practical utility of the $\dot{V}O_{2max}$ metric, evidenced by its use for clinical screening, athlete assessment, and determining exercise intensity [2]. Practitioners also use $\dot{V}O_{2max}$ to prognosticate mortality (for every 1 mL^.^ kg^-1.^min^-1^ increase in $\dot{V}O_{2max}$, a 9% reduction of the relative risk of all-cause mortality) [3] and aerobic endurance performance. While $\dot{V}O_{2max}$ is of great importance, the ability to measure a “true" $\dot{V}O_{2max}$ is challenging and methodological approaches are controversial.

The primary criterion for a "true" $\dot{V}O_{2max}$ is the plateau of oxygen uptake ($\dot{V}O_{2pl}$) with increasing work rate, described by A.V Hill as the "flattening of the $\dot{V}O_{2}$-work rate relationship" [4]. Subsequently, the landmark study conducted by Taylor and his colleagues proposed the first standard $\dot{V}O_{2pl}$ identification protocol utilizing a 3–5 days discontinuous step-incremented treadmill exercise [5]. With the advent of breath-by-breath gas exchange technology allowing scientists to collect and analyze the exhaled air instantaneously, there has been a shift from the discontinuous protocol to continuous protocol, favored for their efficiency [6]. However, unlike Taylor’s classical protocol, the continuous ramping protocol is associated with high variability in $\dot{V}O_{2pl}$ incidence rates [7–10]. This variability is in part due to the fact that $\dot{V}O_{2pl}$ is influenced by several factors including sampling interval [11], work rate increment [12], training status [13, 14], disease or illness [6], age [15], and $\dot{V}O_{2pl}$ definitions [11]. This high $\dot{V}O_{2pl}$ variability has spurred others to rely on secondary $\dot{V}O_{2max}$ criteria such as heart rate (HR), blood lactate (BLa), respiratory exchange ratio (RER), and RPE to indicate overall effort and indirectly suggest $\dot{V}O_{2max}$ achievement [16]. However, there are known problems (i.e., highly influenced by the subject inter-variability and reported high false-positive rate) with secondary $\dot{V}O_{2max}$ criteria and their continued use to indirectly confer $\dot{V}O_{2max}$ achievement is questionable [7, 16]. The verification phase is emerging as a valuable addition to cardiopulmonary exercise testing to aid in $\dot{V}O_{2pl}$ assessment and $\dot{V}O_{2max}$ determination [16], but widespread use in research and clinical settings is lacking. Thus, exploring other methods that aid in $\dot{V}O_{2pl}$ observation and $\dot{V}O_{2max}$ determination is worthwhile. One leading cause of low $\dot{V}O_{2pl}$ incidence rate is the insufficient duration of the ramp test, particularly at higher work rates, whereby a steady state may not be achieved before fatigue onset [16]. Individual anaerobic capacity and $\dot{V}O_{2}$ kinetics have been recognized as two critical factors in mediating exercise duration at these high exercise intensities and could be critical components that, if enhanced, may facilitate $\dot{V}O_{2pl}$ observation during cardiopulmonary exercise testing [8, 17, 18].

Completing a high-intensity warm-up, called a priming warm-up before exercise can improve $\dot{V}O_{2}$ kinetics, anaerobic capacity, and skeletal muscle perfusion during heavy-to-severe intensity domain exercise [19–21]. It is within these exercise domains that $\dot{V}O_{2max}$ is achievable. Several studies have observed faster oxygen kinetics and reduced slow component of $\dot{V}O_{2}$ by performing a priming warm-up [19, 22, 23]. As such, a $\dot{V}O_{2pl}$ can plausibly be observed earlier and enable an extended exercise duration for a given work rate. Both factors favorably affect the probability of achieving $\dot{V}O_{2pl}$ and $\dot{V}O_{2max}$. It is thought that the priming warm-up reduces the compulsory contribution of anaerobic metabolism during the physiologic adjustment period by speeding the increase of oxidative phosphorylation indicated by an enhanced overall $\dot{V}O_{2}$ kinetics at the onset of exercise [21]. The anaerobic reserve preserved by enhanced overall $\dot{V}O_{2}$ kinetics can then be tapped to prolong duration within heavy-to-severe intensity domain exercise to observe a $\dot{V}O_{2pl}$.

Based on these early studies, two research groups proposed employing the priming warm-up to aid in $\dot{V}O_{2pl}$ observation during a ramp test. The first study employed a continuous ramp test (1 W every 2 s) and reported a $\dot{V}O_{2pl}$ in all subjects using the priming warm-up (intensity was at 50% of the difference between the GET and WR_max_. When using the normal warm-up (15 W) only half the subjects achieved $\dot{V}O_{2pl}$ [24]. However, this study used an absolute cut-off to define $\dot{V}O_{2pl}$ (< 2.1 mL^.^kg^-1.^min^-1^ between the last two ramp test stages), which could yield high false-positive rates when applied with a continuous ramp test [8, 11, 25]. Moreover, the comparison between visits is clouded because the warm-up duration was substantially different (priming: 6 min at priming intensity and 6 min at 15 W; normal: 3 min at 15 W). The similar ramp test exercise duration and WR_max_ cast further doubt as it is thought that the priming warm-up should increase duration and thus WR_max_. The other study [26] found no difference in $\dot{V}O_{2pl}$ incidence rate between the normal (3 min at 50 W) and priming warm-up (9 min at 50 W followed by 6 min at 90% of the workload at GET and 6 min at 50% of the difference between the GET and WR_max_). However, this study used an inadequate active recovery time (6 min at 50 W) between the priming warm-up and ramp test, leading to an abnormally high blood lactate (BLa) of 7.6 ± 2.7 mmol·L^-1^ before the ramp test. The absence of increased incidence of $\dot{V}O_{2pl}$ may be due to the impaired anaerobic capacity and reduced exercise tolerance due to this pre-exercise acidosis.

It is crucial to fully scrutinize the potential of a priming warm-up as an approach to improve $\dot{V}O_{2pl}$ incidence rate during cardiopulmonary exercise testing, given the high value of knowing a “true” $\dot{V}O_{2max}$. Previous studies have offered varying conclusions and encountered some methodological challenges, which have left the benefits of a priming warm-up before cardiopulmonary exercise testing somewhat unclear [24, 26]. Therefore, the present study aimed to compare two different warm-up protocols (normal vs. priming) on the occurrence of $\dot{V}O_{2pl}$ during a ramp test and subsequent verification phase. We avoid the potential bias caused by poor protocol design from previous studies by controlling the same duration of the normal and priming warm up, involving enough recovery duration evaluated by the BLa measurements, and determining the $\dot{V}O_{2pl}$ with a newer and refined method [25]. We hypothesized that the priming warm-up would induce a greater incidence of $\dot{V}O_{2pl}$ compared to the normal warm-up visit, and the verification phase would facilitate the confirmation of $\dot{V}O_{2max}$.

## Materials and methods

### Participants

This study recruited eleven apparently healthy recreational cyclists with at least 3 years of training history (body mass = 78.63 ± 11.26 kg, height = 181.7 ± 9.5 cm, age = 36 ± 9 y, $\dot{V}O_{2max}$ = 51.0 ± 5.2 mL·kg^−1^·min^−1^). We defined recreationally active as self-reported physical activity congruent with the high domain rating of the International Physical Activity Questionnaire (IPAQ) long-form [27] and an above-average $\dot{V}O_{2max}$ for their age (> 45 mL·kg^−1^·min^−1^ on the bike) [28]. Healthy was free of lower extremity musculoskeletal injury within the past 6 months and not currently taking medications that could affect the cardiopulmonary, vascular, renal, or metabolic systems. We determined that 11 subjects were required to achieve statistical significance (α = 0.05; β = 0.80) in $\dot{V}O_{2max}$ using a two-tailed dependent t-test assuming a mean $\dot{V}O_{2max}$ of 53.6 ± 8.3 mL·kg^-1^·min^-1^ with a strong correlation (0.84) and large effect size (0.97) [14]. The participants were recruited from the local cycling club and university campus between 15 November 2022 and 22 September 2023. Written informed consent was obtained after the risks and benefits of the study were explained to the participants prior to their inclusion in the study. The study was approved by the University at Buffalo Institutional Review Board (#00006527).

### Study design

**Fig 1:**
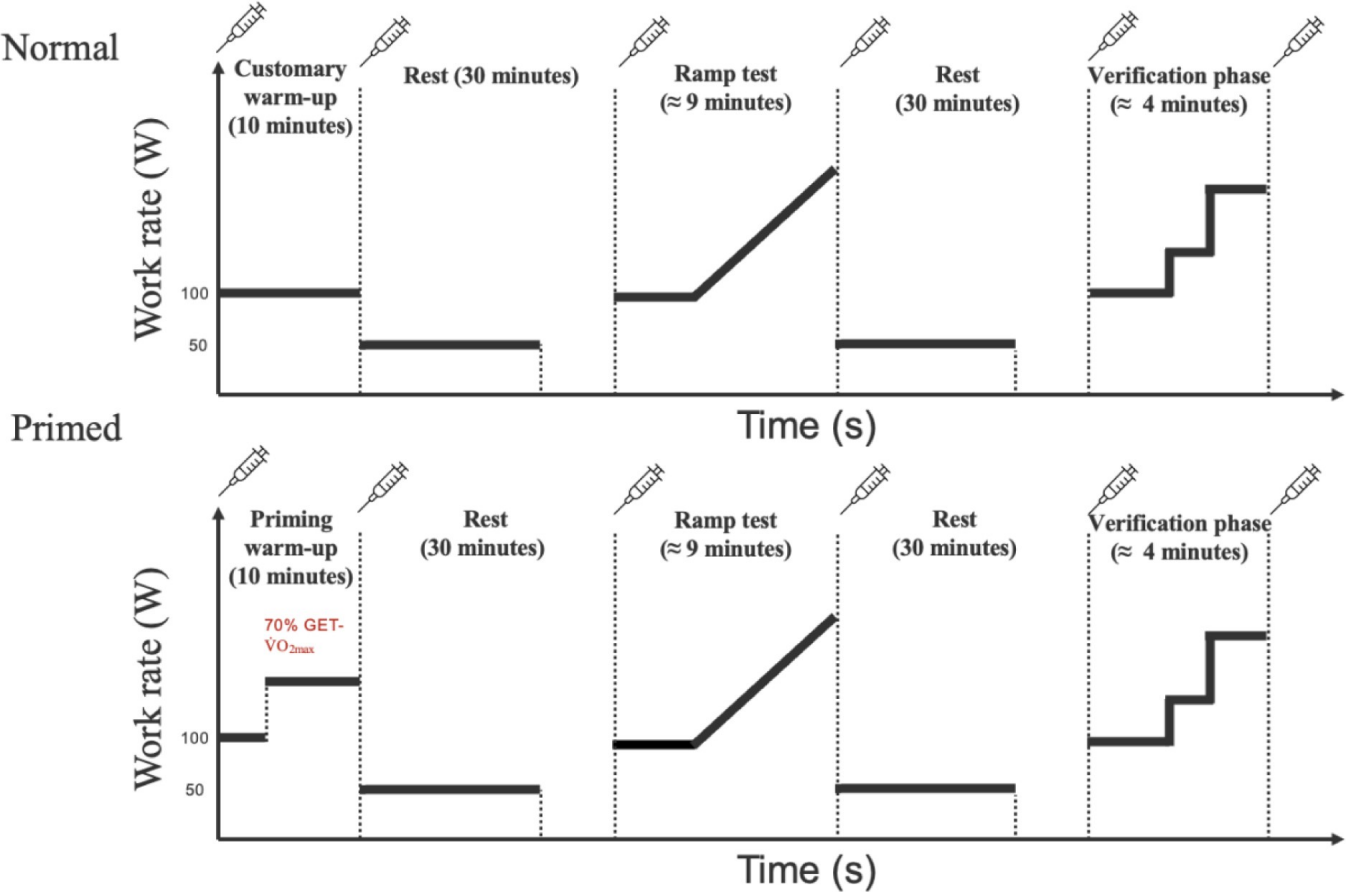
Study procedures for the normal and primed visits.

This research study used a cross-over design whereby participants completed the normal and then primed warm-ups on separate visits (Fig 1). This sequence was required because below-average fitness ($\dot{V}O_{2max}$ ≤ 45 mL·kg^−1^·min^−1^) was an exclusionary criterion and was needed to determine the work rate for the priming warm-up using data from the ramp test in the normal visit. Upon the first arrival of the participants, informed consent was obtained per ethical research standards. Then, participants completed the IPAQ, Participant Information, and Health History forms, followed by height measurement to the nearest X.X cm and body mass to the nearest 0.XX kg (T51P, Ohaus, Pine Brook, NJ) to ensure the study’s eligibility. Afterward, participants completed the normal visit, which was considered our control trial and consisted of a standard warm-up before the ramp test and verification phase. The primed visit was completed on a separate day. It was considered our intervention trial and consisted of a priming warm-up before the ramp test and verification phase. Twenty-four hours before trials, alcohol and physical activity were avoided, and caffeine was not consumed 12 hours before. To avoid the potential influence of diurnal variation on the $\dot{V}O_{2max}$ [29], all subjects completed the trial at the same time of the day (± 1 h). Trials were conducted in temperate, dry conditions (22°C, 40% relative humidity). A urine sample confirmed euhydration (urine specific gravity < 1.025)by a refractometer (Atago, Master-URC/NM, Bellevue, WA) at the beginning of each trial [30].

### Instrumentation

Subjects were instrumented with an HR monitor (T31, Polar, NY, USA) and a one-way breathing mask (Hans Rudolph V2, KS, USA) attached to an indirect calorimeter (TrueOne 2400; ParvoMedics, UT, USA). This device was calibrated before each test according to manufacturer instructions with correction factors for gas and flow within 1%. Using 35 paired samples across a range of exercise intensities, we show very strong reliability measures for $\dot{V}CO_{2}$ (coefficient of variation [CV] = 2.72% with an intraclass correlation coefficient [ICC_2,1_] = 0.998 [95%CI: 0.995–0.999]) and $\dot{V}O_{2}$ (CV = 2.75% with an ICC_2,1_ = 0.996 [95%CI: 0.992–0.998]). Subjects were read a standardized script to familiarize them with the Borg 6–20 ratings of perceived exertion (RPE) scale [31]. Blood lactate (BLa) was measured before and after the warm-up, ramp test, and verification phase by fingertip puncture using an aseptic technique (Lactate Plus, Nova Biomedical, MA, USA).

### Traditional and priming warm-up

The warm-up for both trials were time-matched at 10 min. The traditional warm-up required participants to cycle on an electronically braked cycle ergometer (LC6; Monark Exercise AB, Vansbro, Sweden) for 10 min at 100 W. The priming warm-up consisted of cycling for 4 min at 100 W and then increasing the work rate to 70% of the difference between the GET and $\dot{V}O_{2max}$ (70%Δ) for 6 min [32]. The GET and respiratory compensation point (RCP) were calculated by two trained physiologists during the ramp test in trial 1 using the "V-slope" method [33]. The priming warm-up intensity was calculated as ${WR}_{GET}+0.7\times ({WR}_{max}-{WR}_{GET})$ where ${WR}_{GET}$ stands for the corresponding work rate at GET during the ramp test [32]. Heart rate, RPE, and gas exchange variables (i.e., $\dot{V}O_{2}$, $\dot{V}CO_{2}$, minute ventilation [${\dot{V}}_{E}$,], RER) were gathered throughout the protocol. After the warm-up, a 20-minute active recovery pedaling at a self-selected work rate combined with a 10-minute passive rest period was completed prior to the ramp test. Pilot testing revealed that this biphasic recovery protocol ensured a BLa of 2–3 mmol·L^-1^, which has been suggested to avoid the potential of an impaired anaerobic capacity before the ramp test [21, 34].

### Ramp test

The test began by pedaling at 100 W for 3 min, followed by work rate increases of 30 W·min^-1^ until volitional fatigue or pedal cadence dropped below 60 rpm over 5 sec. Intense verbal encouragement was provided throughout the test. After the ramp test, another 20-minute active recovery followed by a 10-minute passive rest period was completed before the verification phase to avoid potentially impaired anaerobic capacity.

### Verification phase

We employed a biphasic verification phase whereby subjects began cycling at 60% of the ramp test WR_max_ for 2 min, followed by 105% WR_max_ until volitional exhaustion [14] or pedal cadence dropped below 60 rpm. This biphasic verification phase has regularly been used [14, 35] as it reduces the magnitude of a work rate change to 105% (e.g., 105%– 60% = 55%) compared to single phase tests (105%– 0% = 105%) which are thought to extend exercise duration at 105% WR_max_ by mitigating oxygen deficit during the physiological adjustment period.

### Data management

Data were combed to remove spurious values identified by the ROUT method [36]. Breath-by-breath expired gas variables were collected over 30 sec epochs, with the last 30 sec of each stage used for analysis and reporting [11]. $\dot{V}O_{2max}$ was attained if $\dot{V}O_{2pl}$ was observed during the ramp test. We determined $\dot{V}O_{2pl}$ by using an individualized cut-off set at the difference between the actual peak oxygen uptake ($\dot{V}O_{2peak}$) and modeled $\dot{V}O_{2peak}$ if the regression slope is greater than 50% when graphed using the linear portion of the $\dot{V}O_{2}$–work rate relationship [25]. The same method was used to compare the difference between modeled $\dot{V}O_{2}$ and actual $\dot{V}O_{2}$ at the verification phase (105% WR_max_ of the ramp test) to 50% $\dot{V}O_{2}$ slope if the $\dot{V}O_{2pl}$ was absent from the ramp test. The $\dot{V}O_{2max}$ was confirmed by the verification phase if the peak oxygen uptake ($\dot{V}O_{2peak}$) in the ramp test is consistent with the verification phase within 3% [37]. Secondary $\dot{V}O_{2max}$ criteria were defined as HR ≥ 95% of age-predicted HR_max_ (207–0.7·age), BLa ≥ 8 mmol·L^−1^, RPE ≥ 18, and RER ≥ 1.10 [38]. Secondary $\dot{V}O_{2max}$ criteria were considered to be met if 3 out of 4 criteria reached or above the cut-off. $\dot{V}O_{2}$ slope was calculated and expressed by $\Delta \dot{V}O_{2}/\Delta \mathrm{WR}$ across the entire ramp test and each exercise intensity domain (i.e., moderate, heavy, and severe) [39].

### Statistical analysis

All data met assumptions for parametric tests except RCP and RPE, which were not normally distributed. For these variables, the Wilcoxon rank test was performed. We used paired t-tests to compare maximal responses between the normal and primed visits following the warm-up, ramp test, and verification phase and to compare the responses between the verification phase and the ramp test. To control the family-wise error rate, we employed the Holm-Bonferroni correction. Effect sizes were calculated using Hedges g and interpreted as small effect = 0.2, medium effect = 0.5, and large effect = 0.8 [40]. The significance level was 0.05, and analyses were completed using statistical software (GraphPad Prism, v. 9.0.0, Boston, MA, USA).

## Results

Table 1 reports the frequency of $\dot{V}O_{2pl}$, $\dot{V}O_{2max}$ confirmation (via the verification phase), and secondary $\dot{V}O_{2max}$ criteria responses to the ramp test. Table 2 reports the individual peak oxygen uptake ($\dot{V}O_{2peak}$_**)**_ during the ramp test and the verification phase for the normal and primed visits. Subject 1 demonstrated a $\dot{V}O_{2pl}$ in the normal but not in the primed visit, while subject 3 showed a $\dot{V}O_{2pl}$ in the primed but not in the normal visit. All other subjects reported the same frequencies between normal and primed visits. Verification phases confirm all $\dot{V}O_{2max}$ in both visits. No participant demonstrated a $\dot{V}O_{2pl}$ and achieved a higher $\dot{V}O_{2}$ response. However, one participant achieved a 2.4% higher $\dot{V}O_{2peak}$ in the verification phase compared to $\dot{V}O_{2peak}$ in the ramp test without the $\dot{V}O_{2pl}$ occurring during the normal visit.

**Table 1:** Primary and secondary maximal oxygen consumption ($\dot{V}O_{2max}$) criteria frequency.

	$\dot{V}O_{2pl}$	$\dot{V}O_{2max}$ Verification	Secondary Criteria			
			HR	RER	RPE	BLa
Normal ramp test	8/11 (73%)	11/11 (100%)	9/11 (82%)	10/11 (91%)	11/11 (100%)	10/11 (91%)
Priming ramp test	8/11 (73%)	11/11 (100%)	10/11 (91%)	9/11 (82%)	11/11 (100%)	7/11 (64%)

Oxygen consumption plateau ($\dot{V}O_{2}pl$) was determined individually using the methods described by Midgley et al. [25]. $\dot{V}O_{2max}$ confirmed during the verification phase if the actual $\dot{V}O_{2peak}$ from the verification phase was below the modeled $\dot{V}O_{2peak}$. Secondary $\dot{V}O_{2max}$ criteria: HR ≥ 95% of age-predicted HRmax (207 − 0.7 · age), BLa ≥ 8 mmol·L^−1^, RPE ≥ 18, and RER ≥ 1.10 [38]. HR = heart rate, RER = respiratory exchange ratio, BLa = blood lactate concentration.

**Table 2:** Individual absolute peak oxygen uptake ($\dot{V}O_{2peak}$) during the ramp test and the verification phase.

Visits Subjects	Normal			Primed		
	Ramp test	Verification	% Difference	Ramp test	Verification	% Difference
1	4.06^†^	4.01	1.2	4.05	4.04	0.2
2	5.92^†^	5.25	11.3	5.54^†^	5.06	8.7
3	4.16	4.05	2.6	4.11^†^	3.97	3.4
4	3.07	3.00	2.3	3.06	2.77	9.5
5	3.41	3.33	2.3	3.48	3.33	4.3
6	3.89	3.76	3.3	3.90	3.89	0.3
7	3.73	3.71	0.5	3.58	3.59	-0.3
8	3.90	3.63	6.9	3.88	3.45	11.1
9	3.88	3.71	4.4	3.73	3.55	4.8
10	3.38^†^	3.46	-2.4	3.50^†^	3.23	7.7
11	4.67	4.46	4.5	4.74	4.63	2.3

^†^ Oxygen uptake plateau absence. % Difference = percentage difference of $\dot{V}O_{2peak}$ between the ramp test and the verification phase.

**Fig 2:**
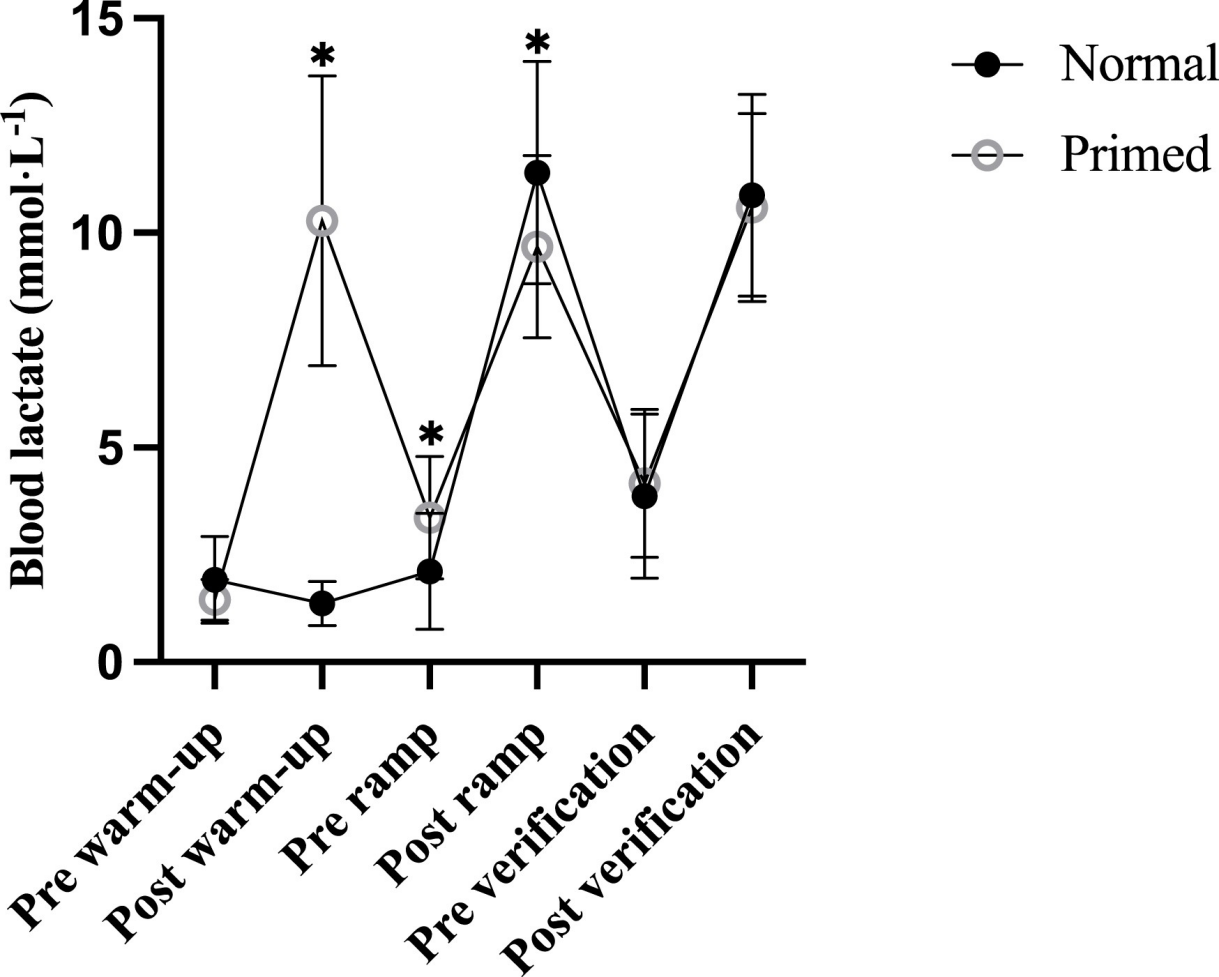
Blood lactate responses throughout the protocol. *p < 0.05 from the normal visit.

As planned, our primed warm-up visit experienced greater physiologic strain compared to the normal warm-up visit as all variables except exercise duration were greater (Table 3), including relative $\dot{V}O_{2}$ (*p* < 0.001, *g* = 4.95), relative $\dot{V}CO_{2}$ (*p* < 0.001, *g* = 6.31), absolute $\dot{V}O_{2}$ (*p* < 0.001, *g* = 5.37), absolute $\dot{V}CO_{2}$ (*p* < 0.001, *g* = 6.70), RER (*p* < 0.001, *g* = 2.47), ${\dot{V}}_{E}$ (*p* < 0.001, *g* = 5.04), HR (*p* < 0.001, *g* = 3.33), percentage of age-predicted HR_max_ (*p* < 0.001, *g* = 3.44), and RPE (*p* = 0.001, *g* = 7.00). Noticeably, 5 out of 11 subjects reached their $\dot{V}O_{2max}$ during the priming warm-up. Fig 2 shows the BLa responses between the normal and primed visits throughout the study. BLa was not different between visits at pre-warm-up (*p* = 0.516, *g* = 0.47, but our priming intervention induced a higher BLa in the primed versus normal vsist at post-warm-up (*p* < 0.001, *g* = 3.70). This difference remained at the pre-ramp test (*p* = 0.005, *g* = 0.90). BLa in the normal visit was higher than the primed visit at the post-ramp (*p* < 0.001, *g* = 2.03). No other differences in BLa were observed.

**Table 3:** Mean physiological responses during the warm-up and maximal physiological responses during the ramp test and the verification phase.

	Warm up		Ramp test		Verification phase	
	Normal	Primed	Normal	Primed	Normal	Primed
Gas exchange variables						
$\dot{V}O_{2}$ (mL·kg^−1^·min^−1^)	20.7 ± 2.2	34 ± 3.1*	51.0 ± 5.2	50.1 ± 4.5	49.6 ± 3.6	47.8 ± 4.0
$\dot{V}O_{2}$ (L·min^−1^)	1.5 ± 0.1	2.7 ± 0.3*	4.0 ± 0.8	4.0 ± 0.7	3.8 ± 0.6	3.8 ± 0.7
$\dot{V}CO_{2}$ (mL·kg^−1^·min^−1^)	17.9 ± 2.1	35.6 ± 3.2*	60.1 ± 4.5	58.2 ± 4.6	59.2 ± 5.1	58.7 ± 5.8
$\dot{V}CO_{2}$ (L·min^−1^)	1.3 ± 0.1	2.8 ± 0.3*	4.7 ± 0.7	4.6 ± 0.6	4.6 ± 0.5	4.5 ± 0.6
RER	0.89 ± 0.04	1.03 ± 0.06*	1.19 ± 0.08	1.17 ± 0.09	1.23 ± 0.09	1.20 ± 0.10^
${\dot{V}}_{E}$ (L·min^−1^)	30.48 ± 4.85	71.76 ± 10.51*	128.0 ± 24.4	125.7 ± 19.2	121.4 ± 20.6	118.3 ± 19.4^
$\dot{V}O_{2}$ Slope (mL·min^−1^·W^-1^)	—	—	10.2 ± 1.0	10.0 ± 1.3	—	—
$\dot{V}O_{2}$ Slope_MOD_ (mL·min^−1^·W^-1^)	—	—	10.2 ± 1.5	10.4 ± 0.9	—	—
$\dot{V}O_{2}$ Slope_HVY_ (mL·min^−1^·W^-1^)	—	—	11.4 ± 2.1	10.4 ± 1.6	—	—
$\dot{V}O_{2}$ Slope_SEV_ (mL·min^−1^·W^-1^)	—	—	7.5 ± 4.5	6.8 ± 5.0	—	—
GET (mL·kg^−1^·min^−1^)	—	—	31.6 ± 4.0	30.8 ± 3.6	—	—
RCP (mL·kg^−1^·min^−1^)	—	—	43.0 ± 5.8	42.9 ± 5.4	—	—
Duration (s)	600	600	570 ± 112	561 ± 90	122 ± 28	120 ± 22
Maximal work rate (W_max_)	100 ± 0	295 ± 44*	356 ± 56	351 ± 44	359 ± 57	362 ± 54
HR (bpm)	110 ± 10	145 ± 11*	180 ± 8	180 ± 8	176 ± 5^	174 ± 6^
Age-predicted HR_max_ (%)	60 ± 5	79 ± 6*	99 ± 4	99 ± 4	95 ± 5^	95 ± 4^
RPE (arbitrary units)	8 ± 1	15 ± 1*	19 ± 1	19 ± 1	19 ± 1	19 ± 1

Data are expressed as mean ± SD. HR = heart rate, Age-predicted HR_max_ = (207 − 0.7 · age), $\dot{V}O_{2}$ = oxygen consumption, $\dot{V}CO_{2}$ = carbon dioxide consumption, RER = respiratory exchange ratio, ${\dot{V}}_{E}$ = minute ventilation, $\dot{V}O_{2}$ slope_MOD_ = $\dot{V}O_{2}$ slope in the moderate exercise intensity domain, $\dot{V}O_{2}$ slope_HVY_ = $\dot{V}O_{2}$ slope in the heavy exercise intensity domain, $\dot{V}O_{2}$ slope_SEV_ = $\dot{V}O_{2}$ slope in the severe exercise intensity domain, GET = gas exchange threshold, RCP = respiratory compensation point, RPE = rating of perceived exertion.

* *p* < 0.05 from the normal visit.

^ *p* < 0.05 from ramp test under the same visit (e.g., primed ramp test vs. primed verification phase).

**Fig 3:**
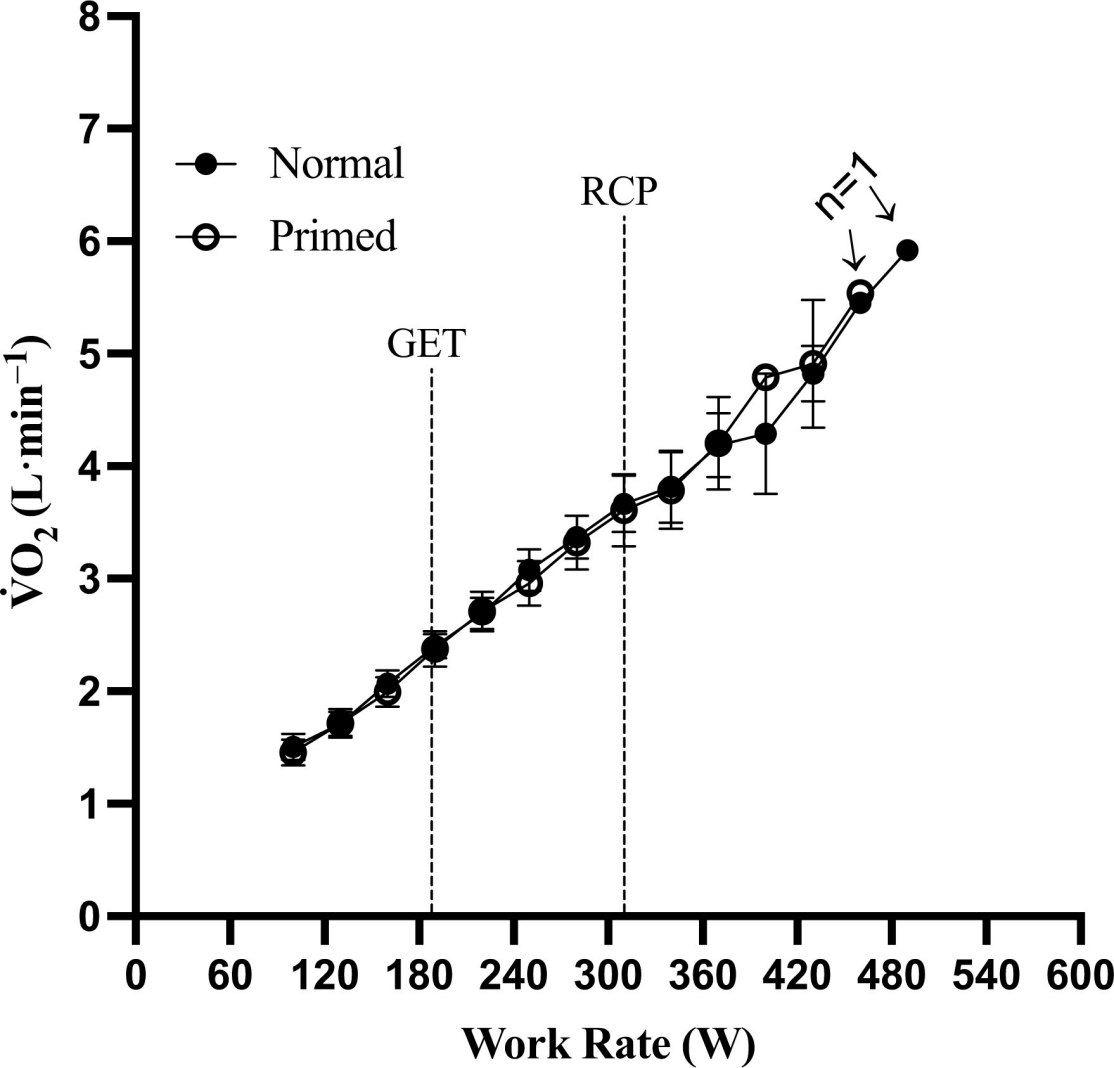
$\dot{V}O_{2}$–work rate relationship during the ramp test. Gas exchange threshold (GET) and respiratory compensation point (RCP) dotted lines represent mean responses.

**Fig 4:**
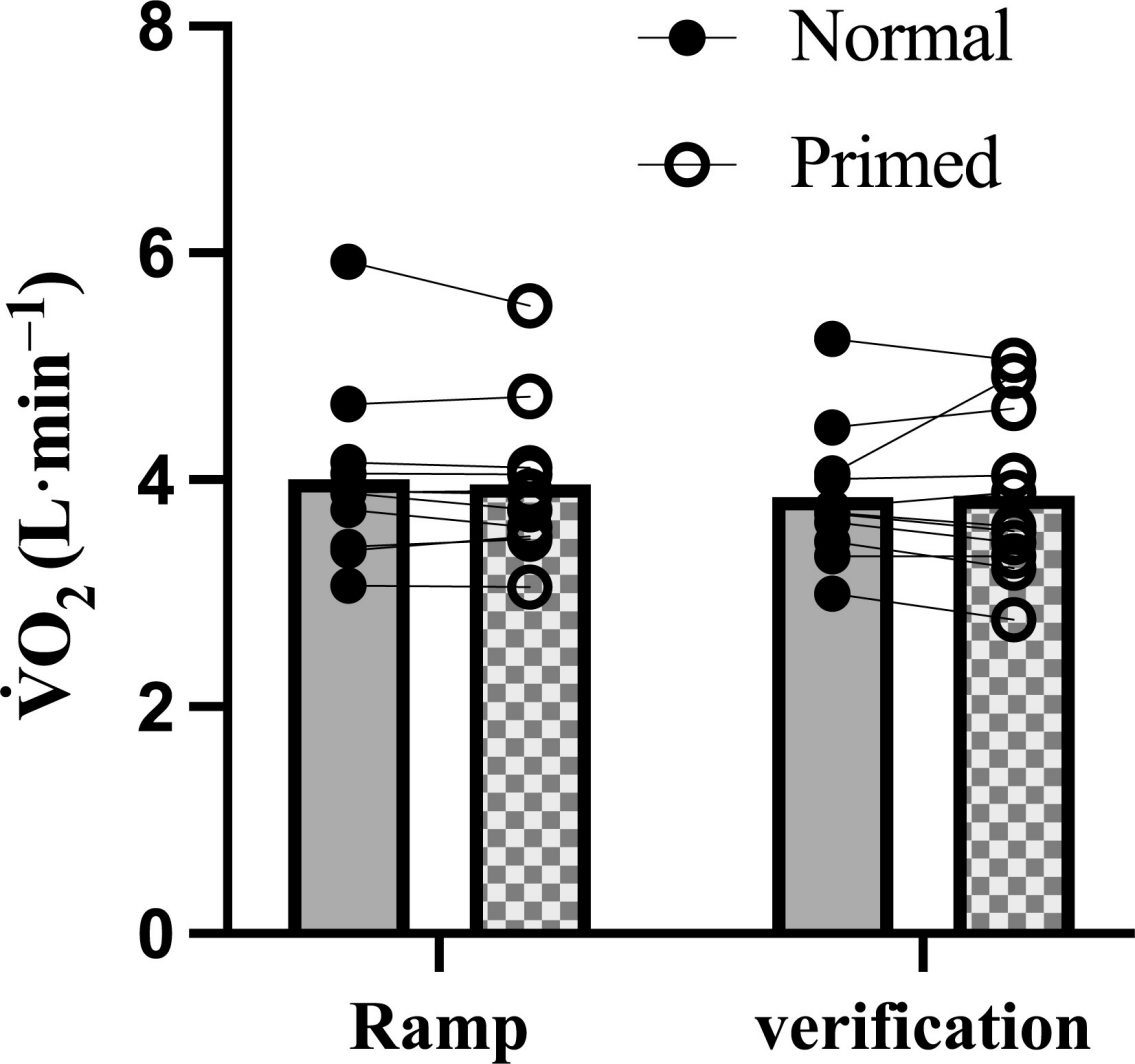
Mean and individual maximal oxygen uptake ($\dot{V}O_{2max}$) in the normal and primed visits.

As displayed in Fig 3 and Table 3, the overall and exercise domain-specific $\dot{V}O_{2}$ slopes were not different between the normal and primed warm-up during the ramp test (*p* ≥ 0.08, g = 0.20–0.56). During the ramp test and verification phase, no differences were observed in any maximal response between visits (all *p* > 0.05; Table 3 and Fig 4). The verification phase relative $\dot{V}O_{2max}$ was lower in the primed visit compared to the normal visit (*p* = 0.05, *g* = 0.49) and primed visit ramp test (*p* = 0.027, *g* = 0.50), but after the Holm-Bonferroni correction, this difference was not significant (α = 0.013). Baseline body mass trended toward significance between visits (*p* = 0.052, *g* = 0.05) as three subjects experienced a 1.4, 1.8, and 2.1 kg body mass gain, respectively, before the priming warm-up trial, which influenced the relative expression of $\dot{V}O_{2max}$. Comparing the primed ramp test to the primed verification phase ${\dot{V}}_{E}$ (*p* = 0.011, *g* = 0.38), HR (*p* < 0.001, *g* = 0.90), percentage of age-predicted HR_max_ (*p* < 0.001, *g* = 1.02), and RER (*p* = 0.006, *g* = 0.54) were higher in the primed verification phase compared to the primed ramp test. The percentage of age-predicted HR_max_ (*p* = 0.008, *g* = 0.81) was lower in the normal verification phase compared to the normal ramp test.

## Discussion

We investigated a prior-heavy intensity warm-up, also known as a priming warm-up, on $\dot{V}O_{2pl}$ incidence rate during a ramping test and confirmation of $\dot{V}O_{2max}$ during a verification phase. The present study is one of the first studies to explore responses in experienced recreational cyclists, as well as the first to employ BLa measurements throughout the protocol to confirm mild acidosis, which is considered to be an important premise for the priming effect [21]. In opposition to our hypothesis, our results showed that the priming warm-up did not affect oxygen kinetics nor cardiopulmonary responses to the ramp test, including the $\dot{V}O_{2pl}$ incidence rate. Based on these data, we do not recommend the use of prior-heavy intensity warm-ups before a ramp test in trained cyclists.

Notably, the ramp test incidence rate of $\dot{V}O_{2pl}$ was equal and unexpectedly high in both visits (73%; Table 1), indicating that the priming warm-up does not influence $\dot{V}O_{2pl}$. This outcome diverges from earlier research on the priming warm-up that observed $\dot{V}O_{2pl}$ incidence rates of 50%, 100%, and 83% in unprimed, heavy intensity primed, and severe intensity primed visits, respectively [24]. However, Niemeyer et al. reported much lower $\dot{V}{O2}_{pl}$ incidence of 40% and 35%, comparable rates between unprimed and primed visits, respectively [26]. There are many factors affecting the $\dot{V}O_{2pl}$ incidence rate, which makes the differences among studies challenging to integrate. The use of different $\dot{V}O_{2pl}$ definitions among studies has been shown to evoke high inter-study variability of $\dot{V}O_{2pl}$ incidence [41]. Examining studies employing a priming warm-up on $\dot{V}O_{2pl}$ incidence rate, Gordan et al. utilized an absolute cut-off of Δ $\dot{V}O_{2}$ < 2.1 mL·kg^-1^·min^-1^ [5] and found a 100% incidence rate. This finding may be erroneously high as absolute cut-offs are known to have high false-positive rates [8]. Moreover, numerous studies have criticized this approach as it is influenced by high inter-subject variability in the $\dot{V}O_{2}$ slope [8, 16, 25]. Likewise, Niemeyer et al. developed their own absolute cut-off of 5.0 mL·min^-1^·W^-1,^ which is more conservative than Δ $\dot{V}O_{2}$ < 2.1 mL·kg^-1^·min^-1^ but remains an absolute criterion which is circumstance to high inter-subject variability and false positive issues. Conversely, we employed an individualized $\dot{V}O_{2pl}$ threshold, which is the current best practice as it minimizes false positives by accounting for individual differences in the oxygen kinetics [25]. In sum, differences in the magnitude $\dot{V}O_{2pl}$ incidence rate among studies can be partly explained by inconsistent $\dot{V}O_{2pl}$ definitions. However, our data and another study [26] begin to clarify the issue of whether a priming warm-up improved $\dot{V}O_{2pl}$ incidence rate by showing there were no differences in incident rate between a priming and normal or customary lower intensity warm-up before a ramping exercise test.

Oxygen kinetics are central to the theory linking a priming warm-up to improved $\dot{V}O_{2pl}$ incidence. To keep consistency with previous studies [26, 42–44], we measured the overall and exercise domain specific $\dot{V}O_{2}$ slopes and observed no differences between visits (Table 3 and Fig 4). The current findings are in line with the results reported by Marles et al., who also found no impact on $\dot{V}O_{2}$ slopes during a ramp test with a 35 W·2 min^-1^ work rate increment after utilizing a priming warm-up [44]. Conversely, Boone et al. observed an increased $\dot{V}O_{2}$ slope in the moderate-intensity domain and a decrease in the heavy to severe domain during the ramp test using a 25 W·min^-1^ work rate increment [42]. This finding is consistent with the research conducted by Niemeyer et al., which also reported an impaired $\dot{V}O_{2}$ slope during heavy to severe activity, whereas the study used an individualized work rate increment with one minute in each stage [26]. By contrast, Jones et al. demonstrated an increased $\dot{V}O_{2}$ slope in both heavy to severe exercise domains and overall status during a 25–35 W·min^-1^ work rate incremental ramp test [43]. A notable fact is that the above studies used a short rest period between priming warm-up and ramp test, resulting in a high BLa before the ramp test, ranging from 6.2–8.1 mmol·L^-1^. It is plausible, then, that an impaired anaerobic capacity limited any potential priming effect on $\dot{V}O_{2}$ kinetics [21]. To avoid such a limitation, we used a 30-minute mixed active recovery and resting protocol to ensure a slight whole-body acidosis before the ramp test (BLa = 3.4 ± 1.4 mmol·L^-1^) in accordance with the recommended best practice [21]. Thus, the similar $\dot{V}O_{2}$ slope in both visits in the current study is unlikely caused by inadequacies in method design, and the absence of a priming effect on the $\dot{V}O_{2}$ slope remains unclear. It is possible that the priming effect, featured by an improved time constant during constant work rate exercise, may not be transferable to incremental work rate exercise hypothesized as an improved $\dot{V}O_{2}$ slope, regardless of the successful implementation of the priming warm-up [45]. The priming warm-up induced acceleration of $\dot{V}O_{2}$ kinetics is thought to be due to an increased cardiodynamic (Phase I) and especially primary (Phase II) amplitude of the $\dot{V}O_{2}$ response with a lower slow component of $\dot{V}O_{2}$ uptake in the last phase (i.e., Phase III) [21, 46]. We used an aggressive work rate increment (i.e., 30 W·min^-1^) during the ramp test and we roughly estimated that only 68% of the Δ $\dot{V}O_{2}$ would occur before the subsequent stage, which may not leave enough time to observe differences in $\dot{V}O_{2}$ slope [6]. To avoid this challenge would require stage durations ≥ 2 min, which we opted against as maintaining exercise intensities where $\dot{V}O_{2pl}$ may be observed for this duration is problematic. One approach we and others have attempted to address this shortcoming during ramp tests is to group $\dot{V}O_{2}$ slope responses within exercise domains to extend the measurement window. However, the time constant and $\dot{V}O_{2}$ slope are two distinct variables indicating different cardiopulmonary responses during exercise. More specifically, the time constant represents the rate at which $\dot{V}O_{2}$ adjusts to a new steady state, indicating muscle oxygen utilization kinetics, while $\dot{V}O_{2}$ slope is the rate of $\dot{V}O_{2}$ response to increasing work rate, which implies the effectiveness of oxygen delivery to meet the energy demand of exercising muscle [47]. The frequent exercise intensity increases characteristic of continuous ramp tests may then obviate any potential observable changes in these variables explaining why we and others could not confirm oxygen kinetics changes. Furthermore, the lack of a priming warm-up induced change in $\dot{V}O_{2}$ slope may simply be due to the fact that the very nature of a continuous ramping protocol may serve as a sort of priming exercise [48]. If the $\dot{V}O_{2}$ slope component of oxygen kinetics cannot be reliably confirmed during continuous ramp tests, then the efficacy of the priming warm-up to improve the $\dot{V}O_{2pl}$ incidence rate may require other approaches such as discontinuous ramping protocols or examination of other VO2 kinetic components to support implementation.

Emerging evidence from a study conducted at the same time as the present study but in another lab showed that a heavy intensity domain priming warm-up did not influence $\dot{V}O_{2}$ slope but showed enhancements in MRT, faster time to $\dot{V}O_{2max}$, and longer $\dot{V}O_{2pl}$ duration compared to control visit [48]. These data indicate that a priming warm-up can potentially improve the highly clinically relevant $\dot{V}O_{2pl}$ incidence rate as we originally hypothesized. However, the $\dot{V}O_{2pl}$ incidence rate was not measured in their study. While both studies employed the same work rate increment (30 W·min^-1^) during the ramp test, a notable difference between studies is the involvement of a three-step transition exercise inclusive of the priming warm-up (i.e., 6 min at 20 W follow by 6 min at 80 W or heavy intensity domain based on condition, with a final 6 min at 20 W before an immediate ramp test). This time efficient step-ramp-step protocol allowed for the individual identification of work rate during the subsequent priming warm-up visit which may have been a more accurate intensity prescription than ours (70% of WR_max_−GET) to induce the priming effect [48]. Initial work rates of the ramp test also differed (20 W vs. 100 W); with the lower starting work rate allowing for measurement of MRT which was faster following the priming warm-up [48]. The speeding of the MRT originated in the light to moderate intensity domain during the ramp test [48] whereas our initial ramp test work rate (100W) was far surpassed this window of observation. Therefore, the primed physiological benefits may not be evident when utilizing a high work rate ramp test with an aggressive work rate increment. Therefore, a priming warm-up conducted like Mariari et al can improve a component of VO2 kinetics leading to a longer duration at work rates showcasing VO2pl, but future research is needed to determine whether these enhanced responses lead to higher VO2pl incidence rates.

Absolute $\dot{V}O_{2max}$ was not different between the ramp test and verification phase, regardless of the warm-up. The verification phase confirmed $\dot{V}O_{2max}$ in 100% of subjects for both visits, respectively. This is an increase from 73% if solely using a ramp test, which indicates an increased 27% confirmation rate and justification of verification phase implementation regardless of warm-up type. Importantly, the average verification phase duration at 105% WR_max_ in the two visits was over 100 sec (normal: 122 ± 28 s vs. primed: 120 ± 22 s), indicating it is unlikely that subjects prematurely terminated the trial before the steady state of $\dot{V}O_{2max}$ was achieved [49]. It is thought that the priming effect could last at least 45 min [50]; as such, its potentiation effects could be diminished during the verification phase, but this was not the case. In the primed verification phase RER was higher and ${\dot{V}}_{E}$ lower compared to the primed ramp test which indicates greater reliance on bicarbonate buffering. This was likely due to the faster increase in work rate and anaerobic metabolism shift during the verification phase compared to the ramp test. The verification phase confirmed $\dot{V}O_{2max}$ in all participants. As such, an alternative interpretation of our data would be that the verification phase is not necessary since the concordance of $\dot{V}O_{2max}$ values remains high between tests, even if it seems like a robust protocol [51]. We oppose this argument with the fact that if knowledge of a ‘true’ $\dot{V}O_{2max}$ is necessary, such as for diagnostic or prognostic outcomes or to assess the efficacy of an intervention (e.g., the priming warm-up), the use of the verification phase is necessary to add confidence that the highest measured $\dot{V}O_{2}$ was indeed the $\dot{V}O_{2max}$. Additionally, the verification phase can be used to identify cases wherein the GXT elicited a submaximal $\dot{V}O_{2}$ response despite the observation of a $\dot{V}O_{2pl}$ (e.g., a false-positive $\dot{V}O_{2pl}$).

The study provided valuable insight into the effect of a priming warm-up on $\dot{V}O_{2pl}$ incidence rate, but limitations may affect the generalizability and interpretation of the findings. First, although the previous findings suggest that the priming effect can last at least 45 min [50], there is uncertainty about whether a 30-min recovery between the warm-up and the ramp test is too long, potentially diminishing any priming effect. However, we believed that the 30-min recovery duration was necessary for BLa to lower to 2–3 mmol·L^-1^ following the priming warm-up and confirm slight acidosis [21]. It is plausible that accumulated fatigue during the priming warm-up visit may have caused the highest $\dot{V}O_{2}$ measured during the verification phase to be lower than the ramp test. Prolonging the rest period before the verification phase or moving it to a subsequent day may partly address this concern. Lastly, if continuous incremental work rate ramp tests occlude the ability to observe changes in $\dot{V}O_{2}$ slope, utilizing a discontinuous step-incremental exercise would be considerable for researchers in future studies.

## Conclusion

Despite evoking physiologic responses consistent with a primed status following the priming warm-up and recovery period, the incidence rate of $\dot{V}O_{2pl}$_,_ oxygen kinetics, $\dot{V}O_{2max}$ measurements were not different between visits. These data indicate that a priming warm up is not useful prior to a ramp test to determine $\dot{V}O_{2max}$ compared to a normal warm up. A verification phase following a ramp test remains a prudent option when knowledge of a ‘true’ $\dot{V}O_{2max}$ is required.

## Supporting information

S1 Data De-identified PRIMING database. (XLSX)
